# Association between serum 25-hydroxyvitamin D [25(OH)D] levels and vaginal delivery in pregnant women: an observational case–control study from Türkiye

**DOI:** 10.3389/fmed.2026.1752714

**Published:** 2026-03-26

**Authors:** Halime Selen, Nurten Özçalkap, Egehan Bilen

**Affiliations:** 1 Department of Nutrition and Dietetics, Faculty of Health Sciences, Ağrı İbrahim Çeçen University, Ağrı, Türkiye; 2 Department of Nutrition and Dietetics, Faculty of Health Sciences, Mardin Artuklu University, Mardin, Türkiye; 3 Department of Midwifery, Faculty of Health Sciences, Ağrı İbrahim Çeçen University, Ağrı, Türkiye; 4 Women’s Health and Obstetrics Clinic, Ağrı Training and Research Hospital, Ağrı, Türkiye; 5 Women’s Health and Obstetrics Clinic, Buca Seyfi Demirsoy Training and Research Hospital, İzmir, Türkiye

**Keywords:** 25-hydroxyvitamin D [25(OH)D], cesarean delivery, fertility, obesity, pregnancy, vaginal delivery

## Abstract

**Objective:**

This study aimed to evaluate the relationship between maternal serum 25-hydroxyvitamin D [25(OH)D] levels and the likelihood of vaginal delivery.

**Materials and methods:**

This observational case–control study was conducted with a total of 298 women, comprising a case group of those who were unable to deliver vaginally and underwent emergency cesarean section (*n* = 49), and a control group of those who delivered vaginally (*n* = 249). The study data were collected using a questionnaire developed in line with the relevant literature. To explore the effects of independent variables on the dependent variable, both univariate and multivariate logistic regression analyses were performed.

**Results:**

According to the findings of our study, higher serum 25(OH)D levels were significantly associated with a reduced risk of cesarean delivery (*β* = −0.369, *p* = 0.001). Specifically, each 1 ng/mL increase in serum 25(OH)D levels reduces the risk of cesarean delivery by approximately 30.8% [Exp(*β*) = 0.692, 95% CI = 0.611–0.783]. On the other hand, a high body mass index (BMI) pre-pregnancy and at the time of delivery, excessive gestational weight gain, and the presence of chronic diseases were associated with a higher risk of cesarean delivery (*p* < 0.05). In addition, giving birth at a later gestational week was found to be associated with a higher rate of vaginal delivery (*p* < 0.05). No significant associations were found between the mode of delivery and maternal age, total number of pregnancies, previous vaginal delivery, pre-pregnancy family planning method, neonatal birth weight, the total number of non-stress tests (NSTs) performed during pregnancy, or the number of antenatal physician visits (*p* > 0.05).

**Conclusion:**

Higher serum vitamin D levels were associated with an increased likelihood of vaginal delivery. In this context, optimizing vitamin D status through sunlight exposure, nutrition, or supplementation may represent an important step in protecting maternal and neonatal health.

## 1 Introduction

Although pregnancy and childbirth are physiological processes for women, the successful completion of vaginal delivery is influenced by various maternal factors (1). While research on how to best support vaginal delivery is steadily increasing, clear and universally accepted evidence on this issue remains limited (2). The factors affecting vaginal delivery can be classified into two groups: non-modifiable factors, such as age, parity, previous mode of delivery, obstetric complications, gestational age, and geographical region, and modifiable factors, such as nutritional habits, gestational diabetes mellitus (GDM), preeclampsia, level of physical activity, use of epidural analgesia, and obesity (2).

Vaginal delivery is associated with more favorable outcomes for maternal and neonatal health compared with cesarean delivery (3, 4). Women who deliver vaginally experience faster recovery and a lower risk of infection and hemorrhage compared with those who undergo cesarean section (5, 6). In a prospective cohort study with 50 years of follow-up evaluating the effects of delivery mode on maternal mortality and long-term risks, mothers who gave birth via cesarean section were found to have an increased risk of death in the long term, particularly due to cardiovascular disease, diabetes, respiratory disorders, and kidney disease (7). Neonates delivered vaginally were observed to experience fewer respiratory difficulties, develop a stronger microbiota profile, and establish earlier bonding with their mothers through enhanced physical contact at birth compared with those born via cesarean section (8–10). Furthermore, a study including more than 490,000 mothers and children reported that children delivered via cesarean section were more susceptible to conditions such as metabolic disorders, infections, and eczema up to the age of five (11).

For all of these reasons, identifying modifiable risk factors that support vaginal delivery and reduce the need for cesarean sections is of clinical importance. In this context, the aim of this study was to evaluate the independent association between maternal serum 25-hydroxyvitamin D [25(OH)D] levels and mode of delivery after accounting for potential obstetric and sociodemographic confounding factors.

## 2 Materials and methods

### 2.1 Study period, design, and setting

This observational case–control study was conducted between 1 March 2025 and 31 August 2025. The case group consisted of women who were unable to deliver vaginally and underwent an emergency cesarean section, while the control group included women who delivered vaginally. The study was carried out in the maternity and gynecology wards of Ağrı Training and Research Hospital, located in the province of Ağrı, Türkiye. All participants provided both verbal and written informed consent prior to participation.

### 2.2 Study population and sample

The study population consisted of women who gave birth at Ağrı Training and Research Hospital during the specified period. The sample comprised data obtained from all voluntary women who met the inclusion criteria determined during the study period and agreed to participate. Participants were consecutively enrolled without any matching. Consequently, the study sample consisted of 298 women, of whom 249 had vaginal deliveries, and 49 underwent emergency cesarean sections. A *post-hoc* power analysis was conducted to assess whether the sample size and the observed differences between the groups would provide sufficient statistical power and control for type I error. The analysis was performed using G*Power-3.1.9.2 software. With 249 participants in the vaginal delivery group and 49 in the emergency cesarean section group, and at an *α* level of 0.05, the effect size was calculated as 1.0877, resulting in a *post-hoc* power of 0.99. The minimum power value required for post-hoc analysis was 0.67. These results indicate that the power analysis is satisfactory and that both the total and group-specific sample sizes are adequate for the study.

### 2.3 Inclusion criteria

Participants were eligible if they met all of the following:

- Age ≥ 18 years
- Delivered a singleton live infant at Ağrı Training and Research Hospital during the study period
- No maternal or neonatal complications following delivery
- Serum 25(OH)D levels measured as part of routine blood tests within 12 h prior to delivery and recorded in the e-health system
- Did not opt for cesarean delivery in the absence of contraindications for vaginal delivery (i.e., not an elective cesarean)
- No history of previous cesarean delivery
- No history of smoking or alcohol consumption

### 2.4 Data collection tools

The study data were collected using a questionnaire developed by the researchers based on a review of the literature. The questionnaire included items on maternal characteristics [age in years, educational status, occupation, income level, place of residence, height (m), pre-pregnancy body weight (kg), body weight at delivery (kg), gestational weight gain (kg)], serum 25(OH)D level (nq/mL), presence of chronic diseases [including diabetes mellitus (DM), hypertension (HT), and hypothyroidism], obstetric history [total number of pregnancies, number of previous vaginal deliveries, and family planning (FP) method used before pregnancy], gestational week at delivery, and neonatal outcomes [birth weight (g), Apgar scores at 1 and 5 min]. Additional data included the total number of non-stress tests (NSTs) performed during pregnancy, excluding the last 24 h prior to delivery, total number of antenatal physician visits during pregnancy, and mode of delivery (vaginal or emergency cesarean section).

Information on participants’ educational status, occupation, income level, place of residence, height, pre-pregnancy body weight, body weight at delivery, presence of chronic disease, total number of pregnancies, number of previous vaginal deliveries, family planning (FP) method used before pregnancy, total number of non-stress tests (NSTs) during pregnancy, and total number of antenatal physician visits was obtained through face-to-face interviews. Body mass index (BMI = kg/m^2^) was calculated using participants’ self-reported body weight and height and classified according to the WHO criteria as underweight (<18.5 kg/m^2^), normal (18.5–24.9 kg/m^2^), overweight (25.0–29.9 kg/m^2^), and obese (≥30 kg/m^2^) (12).

Data on participants’ age, serum 25(OH)D level at delivery, gestational week at delivery, neonatal birth weight, neonatal Apgar scores at 1 and 5 min, and mode of delivery were obtained from patient files and the e-health system. Serum 25(OH)D levels were measured using the immunoassay method [Beckman Coulter Company, California, United States; Catalog No: A98856—Access 25(OH) Vitamin D Total Kit for UniCel DxI System] at the respective healthcare facility. Serum 25(OH)D levels between 30 and 100 ng/mL were considered within the normal reference range. Given that the half-life of serum 25(OH)D ranges between 2 and 3 weeks (13), it was assumed that short-term dietary intake or physical activity in parturient women did not significantly affect these levels.

### 2.5 Ethical considerations

For the conduct of the study, ethical approval was obtained from the Scientific Research Ethics Committee of Ağrı İbrahim Çeçen University (Approval No. 22, dated 30.01.2025). In addition, the study was conducted in accordance with the Declaration of Helsinki.

### 2.6 Statistical analysis methods

The data were initially analyzed using DataBeeg 1.0, an AI-powered software developed in Türkiye in 2023 that supports qualitative, quantitative, and mixed-method analyses. For methodological validation, the results were subsequently cross-checked with SPSS software version 27.0 (IBM Corp., Armonk, NY, United States). The findings obtained from both software packages were convergent and mutually supportive. Descriptive statistical methods—including frequency, percentage, minimum–maximum values, mean, and standard deviation—were used to evaluate the data. The normality of the distribution was assessed with Q–Q plots (14), and a normal distribution was considered when skewness and kurtosis values were within ±3 (15). The chi-square (χ^2^) test was applied to examine the association between categorical variables. For continuous variables, differences between two groups were compared using the independent samples *t*-test. To explore the effects of independent variables on the dependent variable, both univariate and multivariate logistic regression analyses were conducted. Logistic regression was applied when the dependent variable was binary. In this analysis, the probability of the dependent variable belonging to a specific category (e.g., “1 = present” or “0 = absent”) was estimated based on the values of the independent variables. The significance of the independent variables in the model was assessed using the Wald test. The overall fit of the model was evaluated using the Omnibus test and the Hosmer–Lemeshow goodness-of-fit test, while the explanatory power of the model was determined by the Nagelkerke R^2^ value. A *p* < 0.05 was considered statistically significant.

## 3 Results

The comparison of the categorical characteristics of participants according to mode of delivery is presented in Table 1. A statistically significant difference was found between mode of delivery and the presence of chronic disease, with 96.4% of women who delivered vaginally and 81.6% of those who delivered by cesarean section having no chronic disease (*p* = 0.000). A statistically significant difference was also observed between mode of delivery and the number of previous vaginal deliveries, as 40.6% of women who delivered vaginally and 44.9% of those who delivered by cesarean section had no prior vaginal delivery (*p* = 0.032). Furthermore, there was a statistically significant difference between mode of delivery and the 5-min Apgar score of the newborn, with 100% of neonates born vaginally and 95.9% of those born by cesarean section having a score of 7–10 points (indicating a good condition of the newborn) (*p* = 0.001). A statistically significant difference was found between mode of delivery and pre-pregnancy BMI, with 55.4% of women who delivered vaginally and 34.7% of those who delivered by cesarean section having normal pre-pregnancy BMI values (*p* = 0.000). A statistically significant difference was also observed between mode of delivery and BMI at delivery, as 42.2% of women who delivered vaginally were overweight at the time of delivery, whereas 65.3% of those who delivered by cesarean section were obese (*p* = 0.001). No statistically significant differences were found between mode of delivery and participants’ educational status, occupation, income level, place of residence, total number of pregnancies, pre-pregnancy family planning method, or the 1-min Apgar score of the newborn (*p* > 0.05).

**Table 1:** Comparison of categorical characteristics of participants by mode of delivery (*n* = 298).

Variables		Vaginal delivery (*n* = 249)		Emergency cesarean delivery (*n* = 49)		*X* ^2^	*p*
		*n*	%	*n*	%		
Educational status	Not literate	22	8.8	6	12.2	4.192	0.381
	Elementary/middle school graduate	126	50.6	24	49.0		
	High school graduate	55	22.1	14	28.6		
	Associate’s/bachelor’s degree graduate	44	17.7	4	8.2		
	Master’s/PhD degree holder	2	0.8	1	2.0		
Occupation	Housewife	228	91.6	47	95.9	1.089	0.297
	Public/private sector employee	21	8.4	2	4.1		
Income level	Good	61	24.5	10	20.4	0.600	0.741
	Average	169	67.9	36	73.5		
	Poor	19	7.6	3	6.1		
Place of residence	Province	97	39.0	25	51.0	2.465	0.292
	District	70	28.1	11	22.4		
	Village	82	32.9	13	26.5		
Chronic disease status	None	240	96.4	40	81.6	24.331	**0.000**
	Hypothyroidism	4	1.6	1	2.0		
	Diabetes mellitus (DM)	3	1.2	4	8.2		
	Hypertension (HT)	1	0.4	4	8.2		
	DM + HT	1	0.4	0	0.0		
Total number of pregnancies	One	88	35.3	19	38.8	4.083	0.538
	Two	58	23.3	7	14.3		
	Three	40	16.1	9	18.4		
	Four	34	13.7	7	14.3		
	Five	17	6.8	6	12.2		
	Six and above	12	4.8	1	2.0		
Number of previous vaginal deliveries	None	101	40.6	22	44.9	10.548	**0.032**
	One	57	22.9	5	10.2		
	Two	37	14.9	14	28.6		
	Three	30	12.0	2	4.1		
	Four and above	24	9.6	6	12.2		
Pre-pregnancy family planning method	None	221	88.8	46	93.9	4.134	0.530
	Oral contraceptive (OCS)	3	1.2	0	0.0		
	Protective needle	1	0.4	1	2.0		
	Intrauterine device (IUD)	18	7.2	2	4.1		
	Combined oral contraceptive (COC)	3	1.2	0	0.0		
	IUD + COC	3	1.2	0	0.0		
Newborn 1st minute apgar score	7–10 points	244	98.0	46	93.9	5.625	0.060
	4–6 points	4	1.6	1	2.0		
	0–3 points	1	0.4	2	4.1		
Newborn 5th minute apgar score	7–10 points	249	100.0	47	95.9	10.232	**0.001**
	4–6 points	0	0.0	2	4.1		
	0–3 points	0	0.0	0	0.0		
Pre-pregnancy BMI	Underweight	13	5.2	3	6.1	18.589	**0.000**
	Normal	138	55.4	17	34.7		
	Overweight	78	31.3	15	30.6		
	Obese	20	8.0	14	28.6		
BMI at delivery	Underweight	0	0.0	0	0.0	14.880	**0.001**
	Normal	54	21.7	4	8.2		
	Overweight	105	42.2	13	26.5		
	Obese	90	36.1	32	65.3		

BMI, body mass index; COC, combined oral contraceptive; DM, diabetes mellitus; HT, hypertension; IUD, intrauterine device; OCS, oral contraceptive. *p* < 0.05.

The comparison of numerical characteristics of participants by mode of delivery is presented in Table 2. A statistically significant difference was found between pre-pregnancy and delivery BMI values according to mode of delivery, with cesarean section cases having higher BMI values than those who delivered vaginally (*p* = 0.000). In addition, gestational weight gain was statistically higher among women who delivered by cesarean section compared with those who delivered vaginally (*p* = 0.001). A statistically significant difference was also observed between mode of delivery and serum 25(OH)D levels, with women who delivered vaginally having higher serum 25(OH)D levels than those who delivered by cesarean section (*p* = 0.000). Furthermore, a statistically significant difference was found between mode of delivery and gestational week at delivery, with women who delivered vaginally having higher gestational weeks than those who delivered by cesarean section (*p* = 0.001). A statistically significant difference was found between mode of delivery and the total number of antenatal physician visits during pregnancy, with women who delivered by cesarean section attending more visits than those who delivered vaginally (*p* = 0.013). However, no statistically significant differences were observed between mode of delivery and maternal age, neonatal birth weight, or the total number of NSTs performed during pregnancy (*p* > 0.05).

**Table 2:** Comparison of numerical characteristics of participants by mode of delivery (*n* = 298).

Variables	Vaginal delivery (*n* = 249)		Emergency cesarean delivery (*n* = 49)		*t*	*p*
	$\bar{X}$	SD	$\bar{X}$	SD		
Age (years)	25.89	4.79	26.49	6.18	−0.765	0.445
Pre-pregnancy BMI (kg/m^2^)	24.13	4.15	26.48	4.80	−3.532	**0.000**
BMI at delivery (kg/m^2^)	28.51	4.28	32.07	5.04	−5.153	**0.000**
Gestational weight gain (kg)	11.53	5.53	14.33	5.67	−3.225	**0.001**
Serum 25(OH)D level (nq/mL)	11.58	4.98	6.84	3.63	6.332	**0.000**
Birth week	38.90	1.52	38.06	2.22	3.240	**0.001**
Newborn birth weight (g)	3111.96	404.55	3035.10	526.73	1.152	0.250
Number of NSTs performed during pregnancy	4.77	4.48	4.12	3.66	0.952	0.342
Number of antenatal physician visits	7.79	4.22	9.71	7.69	−2.491	**0.013**

25(OH)D, 25-hydroxyvitamin D; BMI, body mass index; NSTs, non-stress tests. *p* < 0.05.

The results of the univariate logistic regression analysis conducted to explain the effect of maternal serum 25(OH)D levels on mode of delivery are presented in Table 3. According to the established model, higher serum 25(OH)D levels were significantly associated with a reduced risk of cesarean delivery (*β* = −0.369, *p* = 0.001). Specifically, each 1 ng/mL increase in serum 25(OH)D level reduces the risk of cesarean delivery by approximately 30.8% [Exp(β) = 0.692, 95% CI = 0.611–0.783].

**Table 3:** Univariate logistic regression analysis of the effect of maternal serum 25(OH)D levels on mode of delivery (*n* = 298).

Variables	β	SH	Wald statistic	SD	*p*	Exp (β)	Confidence interval	
							Lower limit	Upper limit
Constant	1.612	0.512	9.919	1	**0.002**	5.012	–	–
Serum 25(OH)D level	−0.369	0.063	34.208	1	**0.000**	0.692	0.611	0.783

25(OH)D, 25-hydroxyvitamin D. *p* < 0.05.

The results of the multivariate logistic regression analysis conducted to explain the effects of maternal serum 25(OH)D levels and other pregnancy-related characteristics on mode of delivery are presented in Table 4. In all models established, a significant association was observed between serum 25(OH)D levels and mode of delivery, indicating that, as vitamin D levels increased, the risk of cesarean delivery decreased (*p* = 0.000). Higher pre-pregnancy BMI (*β* = 0.107, *p* = 0.006), higher BMI at delivery (*β* = 0.155, *p* = 0.000), excessive gestational weight gain (*β* = 0.093, *p* = 0.003), and the presence of chronic disease (*β* = 2.229, *p* = 0.000) were found to be associated with a higher risk of cesarean delivery. In contrast, higher gestational week at delivery was associated with a reduced risk of cesarean delivery (*β* = −0.212, *p* = 0.039). However, maternal age, total number of pregnancies, number of previous vaginal deliveries, pre-pregnancy family planning method, neonatal birth weight, total number of NSTs during pregnancy, and number of antenatal physician visits were not found to be associated with the ability to deliver vaginally (*p* > 0.05).

**Table 4:** Multivariate logistic regression analysis of the effects of maternal serum 25(OH)D levels and other pregnancy-related characteristics on mode of delivery (*n* = 298).

Model	Variables	β	SH	Wald statistic	sd	*p*	Exp (β)	Confidence interval	
								Lower limit	Upper limit
1	Constant	0.807	1.006	0.644	1	0.422	2.241	-	-
	Serum 25(OH)D level	−0.370	0.063	34.120	1	**0.000**	0.691	0.610	0.782
	Age	0.031	0.034	0.855	1	0.355	1.032	0.966	1.102
2	Constant	−1.161	1.126	1.063	1	0.303	0.313	–	–
	Serum 25(OH)D level	−0.360	0.064	31.931	1	**0.000**	0.698	0.616	0.790
	Pre-pregnancy BMI	0.107	0.039	7.410	1	**0.006**	1.113	1.030	1.203
3	Constant	−3.136	1.316	5.676	1	**0.017**	0.043	–	–
	Serum 25(OH)D level	−0.357	0.065	30.193	1	**0.000**	0.700	0.616	0.795
	BMI at delivery	0.155	0.040	14.797	1	**0.000**	1.167	1.079	1.263
4	Constant	0.464	0.644	0.520	1	0.471	1.590	–	–
	Serum 25(OH)D level	−0.374	0.065	33.279	1	**0.000**	0.688	0.606	0.781
	Gestational weight gain	0.093	0.032	8.590	1	**0.003**	1.097	1.031	1.168
5	Constant	3.801	0.846	20.208	1	**0.000**	44.751	–	–
	Serum 25(OH)D level	−0.389	0.066	34.639	1	**0.000**	0.677	0.595	0.771
	Chronic disease status*	2.229	0.616	13.069	1	**0.000**	0.108	0.032	0.360
6	Constant	1.797	0.619	8.413	1	**0.004**	6.029	–	–
	Serum 25(OH)D level	−0.372	0.063	34.387	1	**0.000**	0.690	0.609	0.781
	Total number of pregnancies	−0.064	0.120	0.284	1	0.594	0.938	0.742	1.186
7	Constant	1.828	0.563	10.524	1	**0.001**	6.222	–	–
	Serum 25(OH)D	−0.376	0.064	34.750	1	**0.000**	0.687	0.606	0.778
	Number of previous vaginal Deliveries	−0.122	0.130	0.875	1	0.350	0.885	0.686	1.143
8	Constant	0.892	0.786	1.290	1	0.256	2.441	–	–
	Serum 25(OH)D	−0.371	0.063	34.264	1	**0.000**	0.690	0.609	0.781
	Pre-pregnancy family planning method*	0.815	0.666	1.499	1	0.221	2.259	0.613	8.329
9	Constant	9.612	3.934	5.971	1	**0.015**	14943.854	–	–
	Serum 25(OH)D level	−0.346	0.063	30.012	1	**0.000**	0.708	0.625	0.801
	Birth week	−0.212	0.103	4.254	1	**0.039**	0.809	0.661	0.990
10	Constant	2.207	1.272	3.010	1	0.083	9.093	–	–
	Serum 25(OH)D level	−0.365	0.063	33.151	1	**0.000**	0.694	0.613	0.786
	Newborn birth weight	0.000	0.000	0.264	1	0.608	1.000	0.999	1.001
11	Constant	1.717	0.542	10.044	1	**0.002**	5.565	-	-
	Serum 25(OH)D level	−0.367	0.063	33.991	1	**0.000**	0.693	0.612	0.784
	Number of NSTs performed during pregnancy	−0.026	0.044	0.358	1	0.549	0.974	0.893	1.062
12	Constant	1.043	0.588	3.148	1	0.076	2.839	–	–
	Serum 25(OH)D level	−0.371	0.065	32.751	1	**0.000**	0.690	0.607	0.783
	Number of antenatal physician visits	0.070	0.037	3.612	1	0.057	1.073	0.998	1.154

*For chronic disease status and pre-pregnancy family planning method, “Present” was used as the reference variable. 25(OH)D, 25-hydroxyvitamin D; BMI, body mass index; NSTs, non-stress tests. *p* < 0.05.

## 4 Discussion

The protection of maternal and child health can be enhanced by promoting vaginal delivery. Nutrition, particularly vitamin D status, plays an important role at all stages of life and is closely associated with pregnancy outcomes (16). In this study, the relationship between maternal serum 25(OH)D levels at delivery and the likelihood of vaginal delivery was investigated.

Our findings indicated that pregnant women with higher serum 25(OH)D levels at the time of delivery were more likely to give birth vaginally. There are a limited number of studies in the literature addressing this issue. In a cohort study evaluating the association between vitamin D intake, serum levels, and fertility, women whose daily vitamin D intake was below the estimated average requirement (EAR) and who had low serum 25(OH)D levels were found to have a lower likelihood of achieving pregnancy, whereas women meeting the EAR level had higher live birth rates (17). In a study including 1,649 pregnant women, those with moderate or severe vitamin D deficiency at delivery were shown to be at a greater risk of cesarean delivery compared with women with sufficient vitamin D levels (18). The same study also reported that women with low vitamin D levels had a higher risk of preeclampsia and preterm birth (18). Another study conducted in Türkiye similarly found that women with low serum vitamin D levels in the first trimester of pregnancy had an increased risk of cesarean delivery (19).

Some studies, while not establishing a direct association with cesarean delivery, have emphasized that low vitamin D levels might lead to adverse perinatal outcomes (20). In a study comparing women who underwent a cesarean section due to dystocia with those who delivered vaginally, the mean serum vitamin D levels were significantly lower in the cesarean group compared with the vaginal delivery group (*p* = 0.02) (21). In the Maternal Vitamin D Osteoporosis Study (MAVIDOS) randomized, double-blind, placebo-controlled trial, which evaluated 965 women who took 1,000 IU of cholecalciferol daily from the 14th week of pregnancy until delivery, the rate of spontaneous vaginal delivery was 65.6% in the vitamin D group and 57.9% in the placebo group, with the difference being statistically significant (*p* = 0.03) (22). A meta-analysis of 5 studies involving 380 pregnant women with GDM reported that vitamin D supplementation reduced the risk of cesarean delivery by 39% (*p* = 0.002) (23). In another study including 253 pregnant women, it was reported that 28% of those with serum 25(OH)D levels below 37.5 nmol/L underwent cesarean delivery, whereas only 14% of those with levels above this threshold did so (24).

Although the results are heterogeneous, understanding the potential effect of vitamin D on vaginal delivery is important. Vitamin D, known as the “sunshine vitamin,” is a steroid-structured vitamin hormone that plays a role in physiological functions such as muscle contraction and calcium absorption (25, 26). The myometrium is composed of smooth muscle tissue that exhibits spontaneous activity, enabling uterine contractions (27). The presence of vitamin D receptors in the myometrium (28–31) provides a biological basis for the effects of this vitamin on the labor process. Similarly, vitamin D receptors have also been identified in the pelvic musculature, which is crucial for cervical dilation during childbirth (32, 33). Through these receptors, vitamin D may directly enhance muscle contraction or indirectly improve muscle efficiency by increasing calcium absorption. Therefore, adequate vitamin D levels may facilitate the labor process through these mechanisms.

In line with the literature, the results of the present study also indicated that the presence of chronic disease, overweight or obesity, or excessive gestational weight gain made vaginal delivery more difficult (34, 35). A retrospective cohort study involving 3,103 pregnant women demonstrated that obesity during pregnancy increased the risk of cesarean delivery by elevating the likelihood of hypertension, GDM, preeclampsia, and macrosomia (36). It is known that the presence of diabetes mellitus and preeclampsia during pregnancy impairs uterine contractility and causes placental insufficiency, thereby complicating vaginal delivery (37, 38). A meta-analysis reported that vitamin D supplementation in pregnant women with preeclampsia reduced the risk of preeclampsia and preterm birth; however, its effect on the mode of delivery was not evaluated (39). Although obese pregnant women exhibit greater uterine contraction intensity compared with underweight women, they have been reported to reach the active phase of labor less frequently and to be less successful in achieving vaginal delivery (40). In a study evaluating pregnant women with a BMI ≥ 29 kg/m^2^, vitamin D supplementation was shown to significantly increase maternal and cord blood vitamin D levels; however, its effect on the mode of delivery was not assessed (41). All of these findings highlight the need for large-scale studies to clarify the potential impact of vitamin D on vaginal delivery. Moreover, they emphasize the importance of collaboration among pregnant women, dietitians, midwives, and gynecologists to maintain optimal body weight through proper planning before and during pregnancy and to manage maternal health conditions effectively.

Within the scope of the “Vitamin D Support Program for Pregnant Women” implemented by the Ministry of Health in Türkiye, pregnant women are provided with free vitamin D supplementation of 1,200 IU/day (9 drops per day) starting from the 12th week of pregnancy until the 6th month postpartum (a total of 1 year) (42). This support is highly important for both maternal and neonatal health (43). A systematic review including 39 studies demonstrated that maternal vitamin D supplementation of ≥400 IU/day reduced respiratory tract infections in children, contributed to growth and bone development, and provided protective effects against neurodevelopmental and autoimmune disorders (44). Although Türkiye is advantageous in terms of the number of sunny days, pregnant women are often unable to achieve adequate sun exposure. Continuing vitamin D supplementation for pregnant women who cannot obtain sufficient sunlight exposure due to socioeconomic or cultural reasons is crucial not only for overall health but also for supporting vaginal delivery.

### 4.1 Limitations and originality of the study

This study had some limitations. The relatively small sample size and its single-center design limited the generalizability of the findings. Participants’ use of vitamin D supplements was not assessed, and body weight was evaluated solely based on self-reports without objective measurements. Additionally, neither regular physical activity habits nor overall nutritional status, including dietary vitamin D intake, were taken into account. In addition, the presence of obstetric complications during pregnancy and whether participants received physiological or psychological support were not investigated. The observational nature of the study design is also insufficient for establishing cause-and-effect relationships. These aspects represent certain methodological shortcomings. Nevertheless, considering the limited number of studies examining the association between serum vitamin D levels and vaginal delivery, the findings of this study may provide valuable contributions to the field. Furthermore, by comprehensively evaluating maternal and neonatal characteristics within the Turkish population, this study emphasizes the public-health significance of vitamin D as a modifiable risk factor for both maternal and neonatal health.

## 5 Conclusion

The findings of this study demonstrated that serum 25(OH)D levels during pregnancy were associated with the likelihood of vaginal delivery. The results indicated that, as maternal serum 25(OH)D levels increased, the probability of achieving vaginal delivery significantly increased. Considering that vaginal delivery yields more favorable outcomes for maternal and neonatal health compared with cesarean section, interventions aimed at maintaining vitamin D levels within the physiological range may influence the mode of delivery.

In this regard, it is important to promote interventions that support maintaining optimal vitamin D levels in pregnant women, such as regular sun exposure, improving dietary habits, and supplementation under medical supervision. However, there remains a lack of sufficient evidence on the most effective approach for sustaining adequate vitamin D levels.

In cases where adequate exposure to sunlight cannot be achieved during pregnancy, it is necessary to raise awareness among pregnant women and encourage the consumption of foods rich in vitamin D. For individuals with identified deficiency, vitamin D supplementation at doses considered appropriate by a physician may provide additional benefits for perinatal outcomes, particularly in the presence of conditions such as preeclampsia or GDM.

In conclusion, it is recommended that vitamin D levels be routinely monitored during pregnancy and that appropriate interventions be implemented in cases of deficiency. However, to better clarify the relationship between vitamin D status and the likelihood of vaginal delivery, further large-sample, multicenter, and longitudinal studies are needed.

## Data Availability

The raw data supporting the conclusions of this article will be made available by the authors, without undue reservation.
